# *Cryphonectria parasitica* Detections in England, Jersey, and Guernsey during 2020–2023 Reveal Newly Affected Areas and Infections by the CHV1 Mycovirus

**DOI:** 10.3390/jof9101036

**Published:** 2023-10-20

**Authors:** Pedro Romon-Ochoa, Pankajini Samal, Caroline Gorton, Alex Lewis, Ruth Chitty, Amy Eacock, Elzbieta Krzywinska, Michael Crampton, Ana Pérez-Sierra, Mick Biddle, Ben Jones, Lisa Ward

**Affiliations:** 1 Forest Research, Plant Pathology Department, Alice Holt Research Station, Farnham, Surrey GU10 4LH, UK; pankajini.samal@forestresearch.gov.uk (P.S.); mick.biddle@forestresearch.gov.uk (M.B.); lisa.ward@forestresearch.gov.uk (L.W.); 2 Forest Research, THDAS-Tree Health Diagnostics and Advisory Service, Alice Holt Research Station, Farnham, Surrey GU10 4LH, UK; caroline.gorton@forestresearch.gov.uk (C.G.); alex.lewis@forestresearch.gov.uk (A.L.); ruth.chitty@forestresearch.gov.uk (R.C.); amy.eacock@forestresearch.gov.uk (A.E.); elzbieta.krzywinska@forestresearch.gov.uk (E.K.); michael.crampton@forestresearch.gov.uk (M.C.); ana.perez-sierra@forestresearch.gov.uk (A.P.-S.); 3 Forestry Commission, 620 Bristol Business Park, Bristol BS16 1EJ, UK; ben.jones@forestrycommission.gov.uk

**Keywords:** chestnut blight, detection, hypovirus, mating types, vegetative compatibility groups

## Abstract

In England, *Cryphonectria parasitica* was detected for the first time in 2011 in a nursery and in 2016 in the wider environment. Surveys between 2017 and 2020 identified the disease at different sites in Berkshire, Buckinghamshire, Cornwall, Derbyshire, Devon, Dorset, London, West Sussex, and the island of Jersey, while the present study comprises the results of the 2020–2023 survey with findings in Derbyshire, Devon, Kent, Nottinghamshire, Herefordshire, Leicestershire, London, West Sussex, and the islands of Jersey and Guernsey. A total of 226 suspected samples were collected from 72 surveyed sites, as far north as Edinburgh and as far west as Plymouth (both of which were negative), and 112 samples tested positive by real-time PCR and isolation from 35 sites. The 112 isolates were tested for the vegetative compatibility group (VCG), mating type, and Cryphonectria hypovirus 1 (CHV1). Twelve VCGs were identified, with two of them (EU-5 and EU-22) being the first records in the UK. Both mating types were present (37% MAT-1 and 63% MAT-2), but only one mating type was present per site and VCG, and perithecia were never observed. Cryphonectria hypovirus 1 (CHV1), consistently subtype-I haplotype E-5, was detected in three isolates at a low concentration (5.9, 21.1, and 33.0 ng/µL) from locations in London, Nottinghamshire, and Devon.

## 1. Introduction

Chestnut blight of *Castanea* species is the main fungal disease affecting chestnut trees both in North America, and in Europe including several outbreaks in England. It is caused by infections of the ascomycete *Cryphonectria parasitica* (Murrill) M. E. Barr. Other major diseases of the same tree hosts are caused by *Gnomoniopsis smithogilvyi* L.A. Shuttlew., E.C.Y. Liew & D.I. Guest (seed and twigs brown rot), *Phytophthora ramorum* Werres, De Cock & Man in ’t Veld (Ramorum decline), *Phytophthora cinnamomi* Rands (root rot), and *Phytophthora cambivora* (Petri) Buisman (ink disease).

Blight-infected trees show top dieback (the tree finishes dying) above cankers on trunk and/or branches and dense epicormic growth below. Other symptoms are orange stromata that appear through swollen lenticels and sometimes internal creamy pale mycelial fans beneath the bark [1]. The fungus originates from Eastern Asia [2] and has little effect on the native host trees (Japanese chestnut *Castanea crenata* Sieb. & Zucc. and Chinese chestnut *Castanea mollissima* Meiling) [3]. 

In contrast, it has caused serious infections of American chestnut (*Castanea dentata* (Marshall) Borkh.) in North America, where it was introduced in the late XIX century initially through the New York Zoo [4], and of sweet chestnut (*Castanea sativa* Mill.) in most continental Europe, where it was first introduced into Italy [5]. The United Kingdom was considered free and protected zone from chestnut blight until 2011 when *C. parasitica* infections were discovered at a nursery farm in Warwickshire, England [6]. This stimulated surveys between 2011–2012, 2013–2015, and 2016 where all the infected trees were destroyed and tighter controls on the import, movement, and export of chestnut trees were introduced [7]. Until 2016, all findings were exclusively in nurseries affecting young trees and, thus, their eradication was easy. However, in December 2016, *C. parasitica* was isolated from four mature trees growing in a car park in Devon and this implied additional Forestry Commission surveys, subsequently diagnosing *C. parasitica* in multiple positive findings in south and mid England [8,9,10].

Two types of spores can be produced, conidia and ascospores, with the latter depending on the presence of both mating-types [11]. Conidia or asexual spores are spread short distances by rain splash and can be produced all year around [12], while ascospores can be ejected longer distances [13]. Dispersal aided by human activities, such as the movement of planting stock, timber, and bark, are pathways of long-distance spread [1]. Bark wounds, such as those produced by hailstorms [14] and the Oriental Chestnut Gall Wasp (OCGW) (*Dryocosmus kuriphilus* Yasumatsu), also present in some areas of England like London [15,16], serve as entry points for this fungus.

Cryphonectria hypovirus 1 (CHV1) is the type member of the family *Hypoviridae* [17]. Hypoviruses are RNA viruses located in the cytoplasm vesicles of their fungal hosts, without a coat protein, and utilizing a double-stranded RNA replication form [18]. Its mode of transmission is through hyphal anastomosis of donor and receptor isolates of the same vegetative compatibility group (VCG) and conidia. Cryphonectria hypovirus 1 acts as a biocontrol agent of chestnut blight in Europe and some parts of North America (Maryland, Virginia, Wisconsin) [13]. In England, this virus was firstly detected in November 2017 [8], and since then, it has been observed in small proportion and low concentration.

In 2020–2023, surveys continued in England and the Channel Islands of Jersey and Guernsey. The tasks of this study were to: (1) follow confirming/refuting the prevalence of *C. parasitica* in symptomatic plant material using real-time PCR and isolation; (2) determine the VCG of each isolate assessing the diversity of the fungus in the area; (3) elucidate their mating type; and (4) screen them for the presence, concentration, and sequence of CHV1.

## 2. Material and Methods

### 2.1. Sampling and Isolation

**Figure 1:**
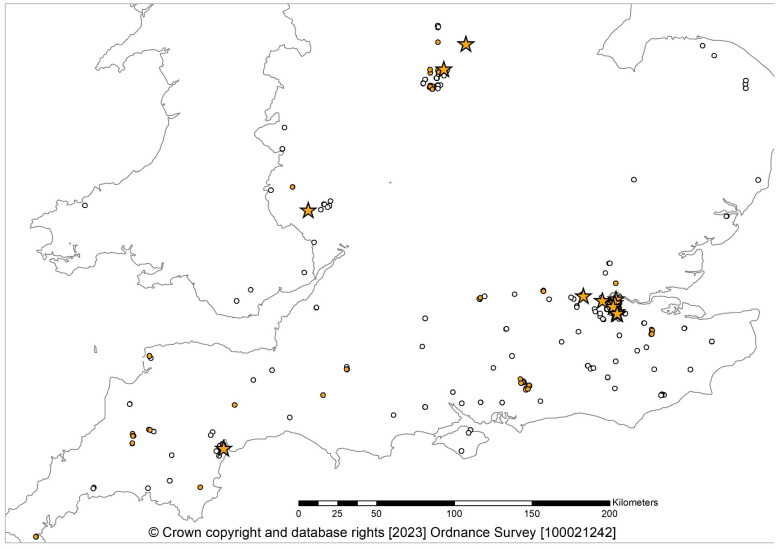
Geographical distribution of sampling sites in England between 2017 and 2023 ([8,10], current study): *Cryphonectria parasitica* positive and CHV1-uninfected (orange circle); *C. parasitica* positive and CHV1-infected (orange star); *C. parasitica* negative (white circles).

New and old sites (Figure 1) were sampled from March 2020 to June 2023. Bark panels (5 × 8 cm) were excised using a chisel to include the margin of each lesion. They were doubled-bagged, labelled with their grid reference coordinates, and sent to the laboratory. Branches with OCGW (15% of total collected samples) galls were also received and analysed because lesions were noted on some galls. In the laboratory, the panels and galls were processed as previously described by Pérez-Sierra et al. [8].

### 2.2. Fungal Detection by Real-Time PCR from Plant Material

A species-specific real-time PCR was performed using a slightly modified version of the TaqMan dual hydrolysis probe assay described by Chandelier et al. [19]. 

Among the modifications, DNA was extracted from each approximate 0.5 g subsample using the DNeasy Plant Pro kit (Qiagen, Manchester, UK). Three homogenisation cycles of 2000 rpm for 20 s each with a dwell time of 5 min between cycles in the PowerLyzer 24 homogeniser (Qiagen) were used. PCRs were carried out on a LightCycler® 480 (Roche, Welwyn, UK). For the assay, a 10 µL reaction volume was prepared, comprising 5 µL of Takyon Blue (Eurogentec, Seraing, Belgium), 0.5 µL (final concentration 500 nM) of the primers [10], and 0.25 µL (final concentration 250 nM) of the probes [10]. Moreover, 2 µL of molecular grade water and 0.5 µL of DNA extract were used.

### 2.3. Vegetative Incompatibility Genes (Vic) Profiles

DNA extracts obtained from the plant material above were used. The alleles at six vic loci were amplified using a modified version of the PCR assays described by Cornejo et al. [20], but without fluorescent primers and thus normal reaction mixtures and normal end-point electrophoresis as previously described [10]. Multilocus genotype profiles were determined, and comparisons were made between the profiles and those stated by Cortesi and Milgroom [21]. 

A normal PCR 96-well plate permitted the processing of eight isolates simultaneously with alternative allele reactions for the six genes vic1a, vic2, vic3a, vic4, vic6, and vic7, respectively, situated in each uneven and even well (12 columns) per row.

The diversity of VCGs was assessed using the Shannon and Wiener index H’, calculated as H’ = −Ʃpi ln pi, where pi is the frequency of the VCG in each county [4].

### 2.4. Mating Types

Mating types were also determined following Cornejo et al. [20] and the same DNA. The reaction mixture was as previously described [10]. At times, if space was available, multiplex PCRs were performed simultaneously with the vic profile generation because the thermocycling conditions were the same (see Section 2.3). Electrophoresis conditions were the same [10].

### 2.5. Cryphonectria Hypovirus 1 (CHV1) Concentration and Sequencing

Even when the concentration of this virus is medium–high—detectable with just toothpick colony reverse-transcription PCR directly from the PDA plates, as outlined by Romon-Ochoa et al. 2022 and references therein—the samples we receive from the wider environment usually contain low loads and no colour change, regardless of the presence or absence of the virus. So, from each isolate, a 5 mm diam. plug was subcultured into 2 mL Eppendorf tubes containing 1 mL of 2% malt extract broth (MEB), maximising the extracted mycelium amount, and processed as previously described by Romon-Ochoa et al. [10].

RNA was then reverse-transcribed and amplified using a one-step reverse transcription polymerase chain reaction (RT-PCR) kit (Qiagen) with the primers stated in Gobbin et al. [22] and the described procedure [10]. Amplicon electrophoresis was the same as for the vic profiles (described in Section 2.3) and quantified with Biorad Gel Doc XR+ band quantification software Image Lab 6.1.0. in comparison with a CSL-MDNA-1 kb ladder (Cleaver Scientific, Rugby, UK). CHV1 298 bp band was quantified.

Amplified products were purified using the DNA clean and concentrator kit (Zymo Research, Cambridge, UK) and sequenced by Source Bioscience, Ltd. (Cambridge, UK). Forward and reverse sequences were aligned, and consensus was determined with SEQUENCHER 5.4.6. BLAST searches were conducted and a dataset encompassing the most up to date hypovirus sequences from GenBank was compiled in MEGA5 [23]. Sequences were aligned online with MAFFT6 [24] using the FFT-NS-i option. The dataset was analysed using maximum likelihood (ML). DNA alignments were converted to PHYLIP format using ALTER [25]. ML analyses were performed using PhyML 3.0 [26], simultaneously determining the substitution model at the ATCG Montpellier Bioinformatics Platform and with 1000 bootstrap replicates in the analysis.

Each virus-infected fungal isolate was preserved by the two methods stated in the work by Romon-Ochoa et al. [27], while the negative isolates were conserved in 15% glycerol in cryotubes at −80 °C.

## 3. Results

### 3.1. Isolations

**Figure 2:**
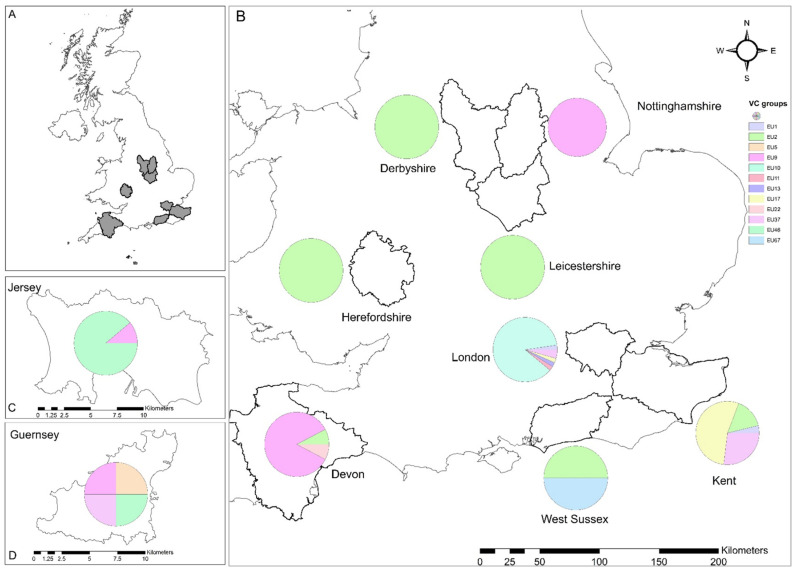
Diversity of vegetative compatibility groups (VCGs) of *Cryphonectria parasitica* (**A**) detected in England (**B**), Jersey (**C**), and Guernsey (**D**). The VCGs are grouped in their respective counties or islands, according to Table 1.

A total of 226 suspected samples were collected from 72 surveyed sites. Chestnut blight was identified at 35 (48% of the surveyed sites) sites: 1 site in Derbyshire, 4 in Devon, 3 in Kent, 2 in Nottinghamshire, 1 in Herefordshire, 1 in Leicestershire, 10 in London, 1 in West Sussex, 9 in the island of Jersey, and 3 in the island of Guernsey (Figure 2, Table 1). The presence of *C. parasitica* was confirmed by molecular and/or culture methods on 112 samples (49% of the suspect samples, Table 1). In total, 20 new positive sites were discovered in addition to 15 sites, which were among those already detected during the previous surveys in 2017–2018 [8] and 2019–2020 [10]. Thirty-seven sites surveyed were negative. Out of 17 twigs suspected to have OCGW galls, 5 tested positive for *C. parasitica* in real-time and culture tests.

### 3.2. Fungal Detections

All samples were tested for the presence of *C. parasitica* using real-time PCR, of which, 112 tested positive; from which, axenic cultures were successfully isolated (Table 1). Most of the other potential canker agents, isolated from the samples that tested negative for *C. parasitica*, based on ITS sequencing, were *Gnomoniopsis smithogilvyi* L.A. Shuttlew., E.C.Y. Liew (61 isolates), *Sirococcus castaneae* (Prill. & Delacr.) J.B. Mey., Senn-Irlet & T.N. Sieber (8), *Pezicula pseudocinnamomea* Chen Chen, Verkley & Crous (8), *Diaporthe rudis* (Fr.) Nitschke (16), *Alternaria infectoria* E.G. Simmons (13), and *Fusarium lateritium* Nees (8 isolates).

### 3.3. VCGs

**Figure 3:**
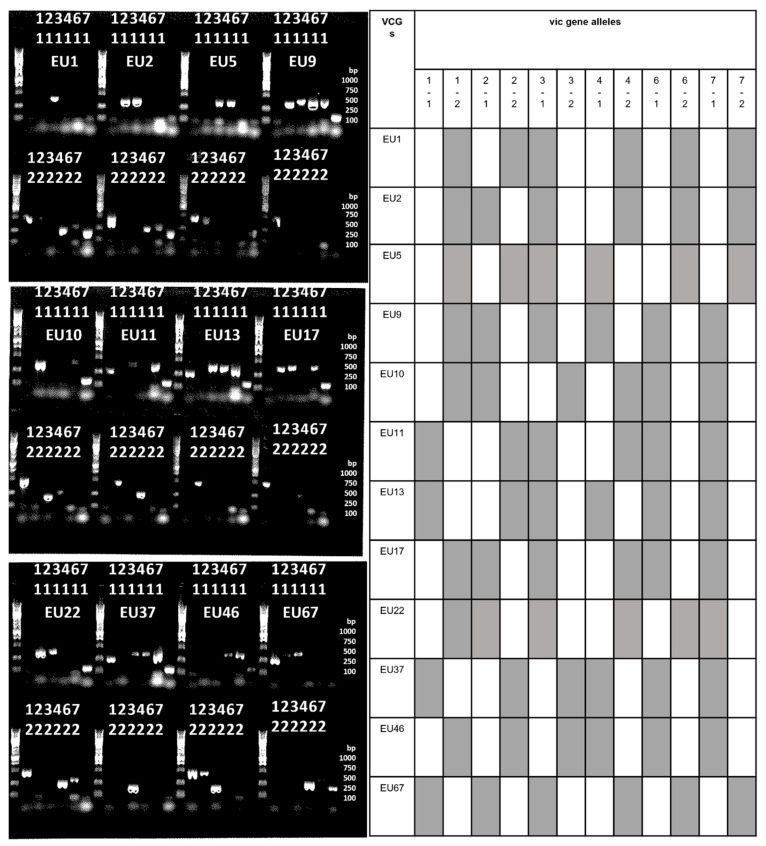
Agarose gel view with the results for the six PCR assays [20] for all the vegetative compatibility *Cryphonectria parasitica* groups that were detected during the present study, with one allele (1) in the upper gel of the pair and the opposite allele (2) in the lower gel of the pair.

The allele profiles obtained for the six vic genes of all the VCGs identified in the present survey are shown in Figure 3. A total of 12 VCGs were identified in this survey (Figure 2, Table 1). The dominant VCGs were EU10 (thirty-nine isolates from London), EU17 (fourteen isolates from Kent, and one from London), EU9 (eleven isolates from Devon, six from Nottinghamshire, one from Jersey, and one from Guernsey), EU2 (four isolates from Kent, three from Derbyshire, three from Herefordshire, one from Devon, one from Leicestershire, and one from West Sussex), EU37 (seven isolates from Kent, two from London, and one from Guernsey), and EU46 (eight isolates from Jersey island and one from Guernsey island).

The EU2 VC group was the most widespread (present in 6/10 counties). Other, relatively minor groups of VCGs were EU1 (one from Kent and one from London), EU11 (one from London), EU13 (one from London), EU22 (one from Devon), EU67 (one from West Sussex), and EU5 (one from Guernsey). European VCGs EU5 and EU22 had not been found in the UK previously. EU5 was found at a new site in Guernsey. The EU22 VCG was identified at an existing infected site, from which only one previous sample had been taken from a different tree that yielded EU12.

Guernsey returned the highest VCG diversity among the surveyed regions, followed by Kent, while Jersey, Devon, London, and West Sussex (ascending order) returned the lowest (Table 1).

### 3.4. Perithecia Occurrence

Both MAT idiomorphs were detected in this population of *C. parasitica*. Of 112 cultures, 41 (37%) were identified as MAT-1 and 71 (63%) as MAT-2 (Table 2). No isolates were amplified for both mating types. Only one mating type was identified at each site per VCG. Perithecia of *C. parasitica* were never observed.

### 3.5. Hypovirus Detection and Sequencing

**Figure 4:**
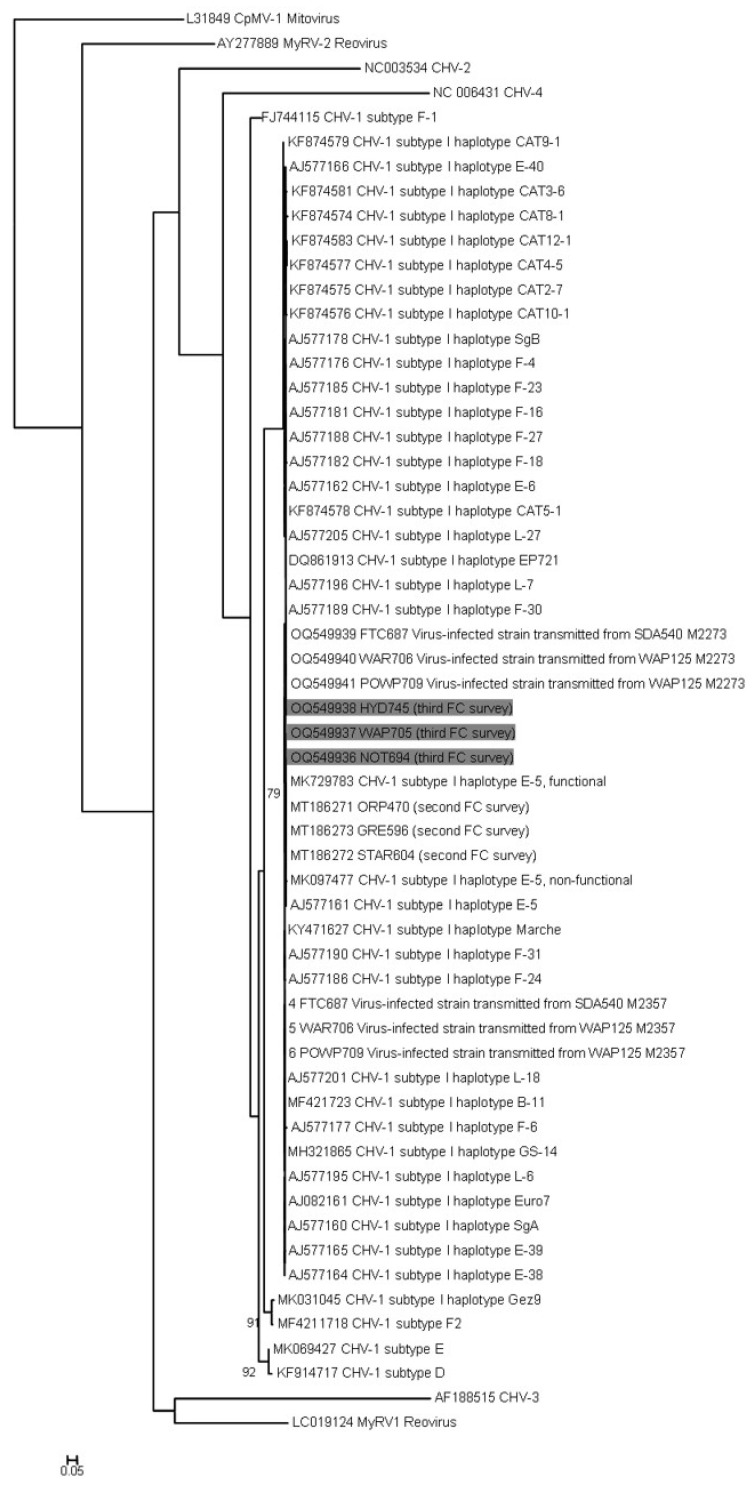
Phylogenetic tree with Maximum Likelihood and Bayesian analyses results of the generated sequences of *Cryphonectria* hypovirus 1 (CHV1) from three isolates (shaded), showing where the sequences match within other CHV1 subtype I haplotypes. Scale bar—total nucleotide differences between taxa.

The 112 isolates were screened for CHV1. The virus was detected in only three of those isolates. One of the positive isolates was isolated from a site in Nottinghamshire (VCG EU9, MAT-1, 21.1 ng/µL), which represents the furthest north that the virus has been detected, and another isolate was from one site in London (VCG EU37, 5.9 ng/µL). CHV1 was detected again in an isolate obtained from Devon (EU9, MAT-2, 33.0 ng/µL) as in a previous study [8]. CHV1 isolates identified during this survey were sequenced (GenBank accession numbers OQ549936–OQ549938). CHV1-positive isolates of *C. parasitica* from the UK clustered with non-mutated subtype-I haplotype E-5 of this hypovirus (Figure 4). In the ML analyses, the best-fitted substitution model was K80+G with a gamma shape parameter of 2.386. Overall, the virus was observed at a low frequency and low concentration.

## 4. Discussion

When compared with the previous two surveys from 2017 to 2018 [8] and 2019 to 2020 [10], no infected trees were detected in Berkshire, Buckinghamshire, Cornwall, or Dorset, concluding that the eradication campaign did not fail there, while sampling efforts rendered new cultures in Kent, Nottinghamshire, Herefordshire, and Leicestershire (totally new areas). Mostly, the situation is not expanding since the cankers are very contained and they are progressing very slowly [8,10].

Twelve *C. parasitica* VCGs were identified in this surveillance campaign across sites in England, Jersey, and Guernsey. There was no major shift from the previous two surveys, despite EU5 and EU22 being recorded for the first time in the area. The previously detected VCGs EU65 [8], and EU12 and EU33 [10], were not observed, although this is not surprising as they only formed a minority of findings when first detected, respectively, in 2017 and 2019. EU10 (35%), EU9 (17%), EU17 (13%), EU2 (11%), EU37 (9%), and EU46 (8%) were the most common VCGs in the population obtained in this study.

Some of the observed VCGs occur across Europe. For example, VCG EU10 is present in Bulgaria, Bosnia, Macedonia, Greece, Italy, and Switzerland [5,28,29,30,31]. VCG EU9 is present in Portugal, Spain, France, and Hungary. VCG EU17 is present in Croatia, Bosnia, Italy, France, Slovakia, Hungary, Switzerland, and some parts of the U.S.A. [32,33,34,35]. Vegetative compatibility group EU2 has been recorded, for example, in Croatia, U.S.A., and in the oaks in Hungary [36].

The *C. parasitica* population observed in this and previous UK studies is among the most diverse populations in Europe, although the diversities of local populations in certain regions such as Nottinghamshire, Herefordshire, London, Devon, and Jersey are smaller. The reduced pattern of disease spread [18], the unsuitability of the weather [37], and the dominance of certain VCGs at several outbreaks are clear indications of several introduction events at different times and from different sources, particularly by the human trade of living plants [8]. Eradication or containment measures are being implemented at affected sites [7]; therefore, it is expected that the diversity of the UK population will reduce because of these measures.

Regarding CHV1, the hypovirus was amplified from two different *C. parasitica* VCGs (EU37 and EU9). The findings of CHV1 in this survey were made from isolates collected in London, Devon, and Nottinghamshire. No positive detections of the hypovirus were made from Derbyshire, despite previous observations in this location [8], which is normal since the Derbyshire population is among one of the most diverse populations globally, showing the highest Shannon diversity index in 2017 [8]. No mycovirus was present in the Channel Islands.

The findings of the hypovirus were always subtype-I haplotype E-5, as previously in England [8,9,10]. Six different subtypes of CHV1 have been characterised in Europe: subtypes I, F1, F2, E, D, and G [38]. CHV1 subtype I, commonly known as the Italian subtype, is the most spread in Europe, and is common in Italy, Bosnia-Herzegovina, Croatia, France, Greece, Macedonia, Slovenia, Switzerland, and Turkey [39,40]. It causes mild reduced virulence in the chestnut blight fungus and, thus, it is widespread used for biological control in most of Europe. Its presence could be an indicator of how long chestnut blight has been endophytically present in the UK because natural hypovirulence starts to appear several decades after the establishment of a new fungal population [41]. The E-5 haplotype was present in total (among all recent surveys up to date ([8,10], this study) in thirteen English isolates. Its distribution is very poorly studied in general but was described as rare by Gobbin et al. [22], and it has been experimentally introduced for example in a pilot site near Monthey (D. Rigling, per. com.) becoming very established in western Switzerland.

Further research is ongoing to determine the diversity of *C. parasitica*. Indeed, further surveillance efforts are needed in the area located between Leicestershire and London counties. Infected sites are discovered during routine surveillance or reports submitted rather than the presence of clear symptoms or widespread tree decline. Eradication measures have been taken, depending on the practicality of operations and presence of CHV1. Contrary to North America, where the CHV1 spread is highly hampered by the high number of VCGs, research in England is now mainly focused on possible biocontrol using CHV1 on sites where one dominant VCG is present and where CHV1 has been detected, such as London (EU10), Devon (EU9), and Nottinghamshire (EU9). CHV1 from highly infected fungal strains from continental Europe (mainly EU10 and EU9) has been successfully transmitted to uninfected English *C. parasitica* isolates of the same VCGs, and further experiments about the *in planta* behaviour under controlled conditions of these virus-infected isolates have been completed successfully [27,42,43]. This success, along with the evidence of three findings of naturally occurring CHV1 observed in this study, is encouraging, and paves the way for further research into its use as a biocontrol agent in the UK, subject to the relevant field permits and regulatory consent, given that this fungal pathogen is considered a protected-zone quarantine pest in the European Union according to the Commission Implementing Regulation 2019/2072.

In the meantime, further transmissions for Kent (EU17) or Jersey (EU46) will be experimentally achieved. Furthermore, the population structure of the fungus in England is being investigated using haplotyping by sequencing and microsatellites, to discern the exact number of introductions, establish the relatedness of those introductions to neighbouring countries, and identify a possible hidden sexual recombination.

## Figures and Tables

**Table 1:** Vegetative compatibility groups (VCGs), virus-infected isolates of *Cryphonectria parasitica* (shown in parentheses), and Shannon diversity index (H’) of isolates obtained from affected sites (surveyed 2020–2023) in England, Jersey, and Guernsey.

County	No. Positive Sites/Total Sites	No. Molecular Detections	Cultured Isolates		No. Isolates per VCG											H’
				EU1	EU2	EU5	EU9	EU10	EU11	EU13	EU17	EU22	EU37	EU46	EU67	
Derbyshire	1/6	3	3		3											0
Devon	4/8	13	13		1		11 (1)					1				0.51
Kent	3/5	26	26	1	4						14		7			1.06
Nottinghamshire	2/2	6	6				6 (1)									0
Herefordshire	1/1	3	3		3											0
Leicestershire	1/1	1	1		1											0
London	10/16	45	45	1				39	1	1	1		2 (1)			0.57
West Sussex	1/9	2	2		1										1	0.69
Island of Jersey	9/20	9	9				1							8		0.35
Island of Guernsey	3/4	4	4			1	1						1	1		1.38
Total	35/72	112	112	2	13	1	19	39	1	1	15	1	10	9	1	

**Table 2:** Distribution of *Cryphonectria parasitica* mating types (MAT-1 and MAT-2) among the vegetative compatibility groups (VCGs) detected in the 2021–2022 surveyed sites.

VCG	Derbyshire		Devon		Kent		Nottinghamshire		Herefordshire		Leicestershire		London		West Sussex		Island of Jersey		Island of Guernsey	
	MAT-1	MAT-2	MAT-1	MAT-2	MAT-1	MAT-2	MAT-1	MAT-2	MAT-1	MAT-2	MAT-1	MAT-2	MAT-1	MAT-2	MAT-1	MAT-2	MAT-1	MAT-2	MAT-1	MAT-2
EU1					1									1						
EU2	3		1			4			3		1					1				
EU5																				1
EU9				11			6											1	1	
EU10														39						
EU11														1						
EU13														1						
EU17					14								1							
EU22			1																	
EU37					7								2							1
EU46																		8		1
EU67																1				
Total	3	-	2	11	22	4	6	-	3	-	1	-	3	42	-	2	-	9	1	3

## Data Availability

The datasets generated and analysed during the current study are deposited in the FR THDAS repository, and are available upon reasonable request.
